# The Fort McMurray Mommy Baby Study: A Protocol to Reduce Maternal Stress Due to the 2016 Fort McMurray Wood Buffalo, Alberta, Canada Wildfire

**DOI:** 10.3389/fpubh.2021.601375

**Published:** 2021-06-17

**Authors:** Ashley Hyde, Barbara S. E. Verstraeten, Joanne K. Olson, Suzanne King, Suzette Brémault-Phillips, David M. Olson

**Affiliations:** ^1^Faculty of Medicine and Dentistry, University of Alberta, Edmonton, AB, Canada; ^2^Department of Obstetrics and Gynecology, University of Alberta, Edmonton, AB, Canada; ^3^Faculty of Nursing, University of Alberta, Edmonton, AB, Canada; ^4^Department of Psychiatry, McGill University, Montreal, QC, Canada; ^5^Douglas Mental Health University Institute, Montreal, QC, Canada; ^6^Department of Occupational Therapy, Faculty of Rehabilitation Medicine, University of Alberta, Edmonton, AB, Canada; ^7^Departments of Obstetrics and Gynecology, Pediatrics and Physiology, University of Alberta, Edmonton, AB, Canada

**Keywords:** natural disaster, wildfire, pregnancy, prenatal maternal stress, maternal mental health, resilience, developmental origins of health and disease, child development

## Abstract

**Introduction:** Data show that maternal stress triggered by exposure to a natural disaster before, during or just after pregnancy is associated with adverse pregnancy and newborn outcomes. In this paper, the first aim is to describe our efforts to test a simple, low-cost intervention to large numbers of women following a major natural disaster. The second aim is to outline the challenges faced and lessons learned during the execution of this natural disaster study.

**Methods:** The setting was the May 2016 Fort McMurray Wood Buffalo wildfire in northern Alberta, Canada. Women who were pregnant or preconception at the time of the disaster were invited to participate via social media. This prospective cohort study included a randomized controlled trial to test the effectiveness of an expressive writing intervention on the levels of prenatal maternal stress and maternal, birth, and early childhood outcomes. At recruitment and at multiple timepoints postpartum, a battery of questionnaires was administered to evaluate objective and subjective stress exposure to the fire as well as maternal mental health, resilience and its contributing factors as well as infant developmental milestones. Qualitative content analysis of the expressive writing was conducted.

**Discussion:** There is an increasing need to develop effective, wide-spread, rapid, and low-cost interventions to reduce prenatal maternal stress, increase resilience, and improve pregnancy outcomes following a natural disaster. Though analysis of data is ongoing, we highlight the strengths of this study which include strong community participation, rapid recruitment of eligible participants, low-cost intervention and data acquisition, and successful testing of the intervention. We acknowledge the challenges we encountered including the high rate of participant disqualifications or losses due to incomplete collection of online data; evacuation, dispersal, and inconsistent return to homes; and the high levels of stress accumulated post-disaster which led to inability to complete the study. Despite potential challenges, there remains a need for such research amid natural disasters.

## Introduction

Climate change is causing more natural disasters. Their frequent worldwide occurrences wreak havoc upon families, particularly at-risk pregnant women. Hurricanes, floods, and wildfires have been linked with increased rates of preterm birth, low birth weight, and other adverse birth outcomes (1–3). Not only can natural disasters and their subsequent prenatal maternal stress (PNMS) have substantial impact on maternal and birth outcomes, they can also significantly impact child developmental outcomes.

### The 2016 Fort McMurray Wildfire

On May 1st, 2016, a wildfire started in the northern Albertan region of Fort McMurray Wood Buffalo (FMWB). Within 3 days, the fire had massively multiplied in size and approached the town of Fort McMurray and nearby municipalities, forcing a mass evacuation of 88,000 people. This natural disaster became Canada's worst in every respect with an estimated cost of $9 billion (4). Twenty-four hundred buildings (10% of total), mostly family houses, were destroyed. Many structures left intact experienced significant smoke damage or ash contamination (5). During the height of the fire, the air pollution index rose to 38 on a 10-point scale (6–8). The town's water supply was contaminated and the large amount of residual ash and soil contained 19 different toxic metals and compounds reaching levels 20 times above recommended limits (5, 9). Residents were not allowed to return to the city until June 1, and then only limited numbers were permitted access. Over the next 2 weeks, about half of the evacuees returned, and it would take until September for the remainder with intact homes to return (10). It took much longer for those whose homes were destroyed to return, and some never did. Even before the wildfire, many FMWB families were experiencing economic difficulties due to layoffs or decreased working hours owing to lower oil prices and demand affecting community mental health (11). Psychologists and other experts predicted that after families returned to their homes, cases of domestic abuse and interpersonal violence would likely increase due to the stressors and uncertainties brought on by this natural disaster (12).

The Developmental Origins of Health and Disease (DOHaD) theory posits that multiple environmental factors operating on the mother before, during, and after pregnancy and while breastfeeding can influence the development of the child in ways that may favor survival in the short-term but may also compromise health in the longer-term (13). Increasing attention has been paid to the environment and experiences of the mother during pregnancy; consequently, PNMS has become an important subject of research. Animal (14–16) and human (17–19) research on PNMS suggest that exposure of the pregnant or preconception individual to stress is associated with a host of negative maternal health and pregnancy outcomes (e.g., preterm birth, gestational diabetes, preeclampsia, fetal growth restriction) as well as adverse developmental trajectories for the offspring - especially on the neurodevelopmental (20) and metabolic level, such as obesity (21), diabetes (22, 23), and cardiovascular issues (24). In addition, past maternal experiences with depression, exposure to adverse life events prior to pregnancy or environmental stressors in the woman's previous generations are thought to have similar adverse consequences as do immediate stressors (25–27).

Previous research studying pregnant women exposed to sudden-onset natural disasters such as the 1998 Quebec Ice Storm (28), the 2009 Iowa Floods (29), and the 2011 Queensland Floods (30) have attempted to disentangle the effects of maternal objective stress exposure, their cognitive appraisal, and their subjective distress due to the disasters on their own mental health and their children's development. These studies demonstrate significant effects of one or more of these aspects of the mothers' stress experience on maternal mood (29, 31), birth outcomes (32, 33), the cognitive (34, 35), behavioral (36–38), and motor (39, 40) development of their children, as well as immune (41, 42) and metabolic health (43, 44). Many of these outcomes have been shown to be mediated by epigenetic effects, still visible at age 13 (45, 46). The results of these disaster studies often suggest that the sex of the child, or the timing of the stressor *in utero*, moderates the impact of the stressor (32, 40).

Beyond prenatal exposure, stress either before conception or soon after birth may also have programming effects. Because an individual's hypothalamic-pituitary-adrenal (HPA) axis can be disrupted for months or even years after a severe disaster (47–49), maternal exposure to a disaster in the months before conception could conceivably influence the earliest moments of embryonic development (50, 51). As for postnatal effects, although the fetal programming hypothesis is based on the connection between the maternal HPA axis and fetal development via the placenta and umbilical cord, maternal stress may also be directly communicated to the young infant via breast milk and maternal behavior (52–54).

### Allostatic Load and Resilience

Whereas a natural disaster is most often limited in time, its resulting stress factors are not. Traumatic events add a considerable amount of stress to the stress load an individual has amassed over the course of its lifetime. Moreover, a short-term natural disaster is often followed by a host of stressors in its aftermath among which are physical and/or environmental, psychological, social, and financial factors. The wear and tear on the body due to the accumulation of life stressors is called allostatic load (AL) (55, 56). AL is the totality of the stressors acting on the body at any given time, both psychological and physical stressors, and how the body attempts to maintain homeostasis in the face of stress. When a threshold is reached at which the body can no longer cope with the AL, the risk increases to develop numerous disease processes associated with allostatic load including preterm birth, susceptibility to infection, and adverse newborn neurodevelopment (57, 58).

Resilience can be defined at the personal or individual level as well as from a socio-ecological perspective. At the individual level, resilience includes physiological, biological, individual, social, and environmental protective factors (59). It is thought of as a dynamic process, the capacity to bounce back, cope with, and recover from adverse events and trauma in order to maintain health and well-being (60). Psychological resilience is considered a major moderator of the relationship between the experience of trauma and the development of posttraumatic psychopathology (61, 62). Social-ecological resilience on the other hand is defined by Folke et al. (63) as the “capacity to adapt or transform in the face of change in socio-ecological systems, particularly unexpected change, in ways that continue to support human well-being (p. 41)” (63). Resilience can also be interpreted in relation to posttraumatic growth, focussing more on “bounce forward” than “bounce back” after experiencing traumatic events.

Cumulatively, the increased allostatic load due to a natural disaster and consequent uncertainty and disruption of lives, toxic chemical ingestion, and the pro-inflammatory stimulus of pollutants in air, water, and soil have the potential to accumulate to the point where individual resilience is overcome and people lose the ability to cope. In this situation they become vulnerable to adverse health outcomes and disease. Hence stress-related adverse outcomes in the case of pregnancy are the difference between an individual's (or a community's) allostatic load and resilience. Improving outcomes requires reducing allostatic load and/or increasing resilience.

### Interventions to Reduce Allostatic Load or Increase Resilience

Several studies indicate that interventions with highly anxious mothers, including reassurance of the fetus' health using additional ultrasound sessions (64, 65), group prenatal care (66), and prenatal education on preparing for childbirth and motherhood (67), may improve perinatal outcomes. However, although a systematic review found that web-based programs for perinatal mental health appear promising, significant gaps in the literature remain (68). The challenge to which we responded was to provide a simple, inexpensive, timely intervention to a large number of women who had experienced the FMWB disaster while pregnant or shortly preconception.

### Aims

The first aim of this paper is to describe our efforts to develop a protocol to deliver and test a rapid intervention to a large number of women following a disaster. The second goal is to outline the challenges faced and lessons learned during the execution of this natural disaster study. It is important to share how challenges were dealt with because the protocol with modifications has been applied in other natural disaster research to date (Houston's Hurricane Harvey) and may be used in future studies.

As this study had a number of objectives, we conceptualized unique objectives and measures for each of the overall aims of this study.

#### Illuminating the Resilience of Pregnant Women Post-trauma: Qualitative Thematic Content Analysis of an Expressive Writing Intervention

We aimed to determine the extent to which a brief, online expressive writing intervention supports maternal resilience. The research questions guiding this project were: (1) Does expressive writing support resilience, reduce stress, and improve outcomes? (2) What thoughts, feelings, themes, experiences, actions, relationships, and factors do the women write about that reflect aspects of resilience? (3) In their post-writing reflections, how effective do the women find expressive writing to be?

##### Intervention: Expressive Writing

Since the 1980's James W. Pennebaker has developed, tested, and refined a simple intervention to help people deal with stressors utilizing expressive writing (69, 70). Narratives or stories reveal both the individual and collective resilience strategies (71). Short bursts of expressive writing (i.e., 15–20 min) were shown to be sufficient to allow for emotional disclosure, the active ingredient in the intervention (70, 72–74), and to improve biochemical markers of physical and immune functioning as well as other physical health outcomes and healthcare utilization (75–83). Each subject in this study was randomly assigned to one of three treatment groups developed in consultation with J. Pennebaker: (1) the expressive writing group, writing about their innermost feelings (active group), (2) a writing group addressing non-emotional issues of healthy lifestyle (non-expressive writing group), and (3) women who did not receive any writing instructions (control or no-intervention group). Expressive writing accesses innermost thoughts and feelings and is a self-reflective learning activity that allows for review and cognitive processing of what has been written and thereby relieves anxiety and builds resilience (73, 74, 81, 84). The format makes participant involvement easy and convenient, even at a distance (85).

#### Prenatal Maternal Stress, Maternal, and Child Outcomes: Effectiveness of a Post-disaster Writing Intervention

Our second goal was to determine the effect that maternal exposure to the FMWB wildfire had on birth outcomes, maternal psychopathology, and infant developmental outcomes. We also aimed to ascertain the effectiveness of the effective writing intervention and its impact on these same outcomes. We hypothesized that maternal objective stress exposure, cognitive appraisal, and subjective stress from the fires would have significant effects on birth outcomes, maternal psychopathology (at 12 months post-wildfire), and infant outcomes (at 18 months of age). In addition, we posited that the effects of PNMS would be moderated by the timing of maternal exposure to the wildfire (from 6 months preconception to 9 months gestation). Finally, we proposed that the PNMS effects from the wildfire would be significantly greater in the no-intervention group and the non-expressive writing group than in the active expressive writing group and that the strength of the buffering effect of the intervention would vary according to the perinatal timing of exposure to the wildfire as well as the intervention.

## Methods

### Setting and Recruitment

The study was designed as a prospective cohort study with a randomized controlled trial to test the effectiveness of an expressive writing intervention. The Fort McMurray Wood Buffalo region has around 1,250 births per year such that ~1,850 women were estimated to be pregnant or about to conceive at the time of the evacuation. Eligible participants included English-speaking women who were pregnant or within 6 months of conception when evacuated due to the 2016 Fort McMurray Wood Buffalo wildfire. Although it was a requirement that participants were residents of the FMWB region at the time of the wildfire, we recognized that some would not return to the community and indeed were not planning on returning at the time we commenced recruitment, which occurred between November 2016 and October 2018. Therefore, women who had temporarily or permanently moved away from the community of FMWB were still welcome to participate.

Study recruitment occurred primarily via social media platforms such as Facebook using targeted advertising. Additionally, we promoted the study and its potential benefits through television and radio features, which encouraged interested mothers to visit the study website^1^ and join the study. Women with multiple pregnancies or with fetuses diagnosed with congenital anomalies were not eligible to participate while women who did not complete the consent and recruitment questionnaires did not receive the intervention. In addition, mothers who experienced a perinatal death or stillbirth after recruitment were withdrawn from the study such that no further questionnaires were administered.

**Figure 1:**
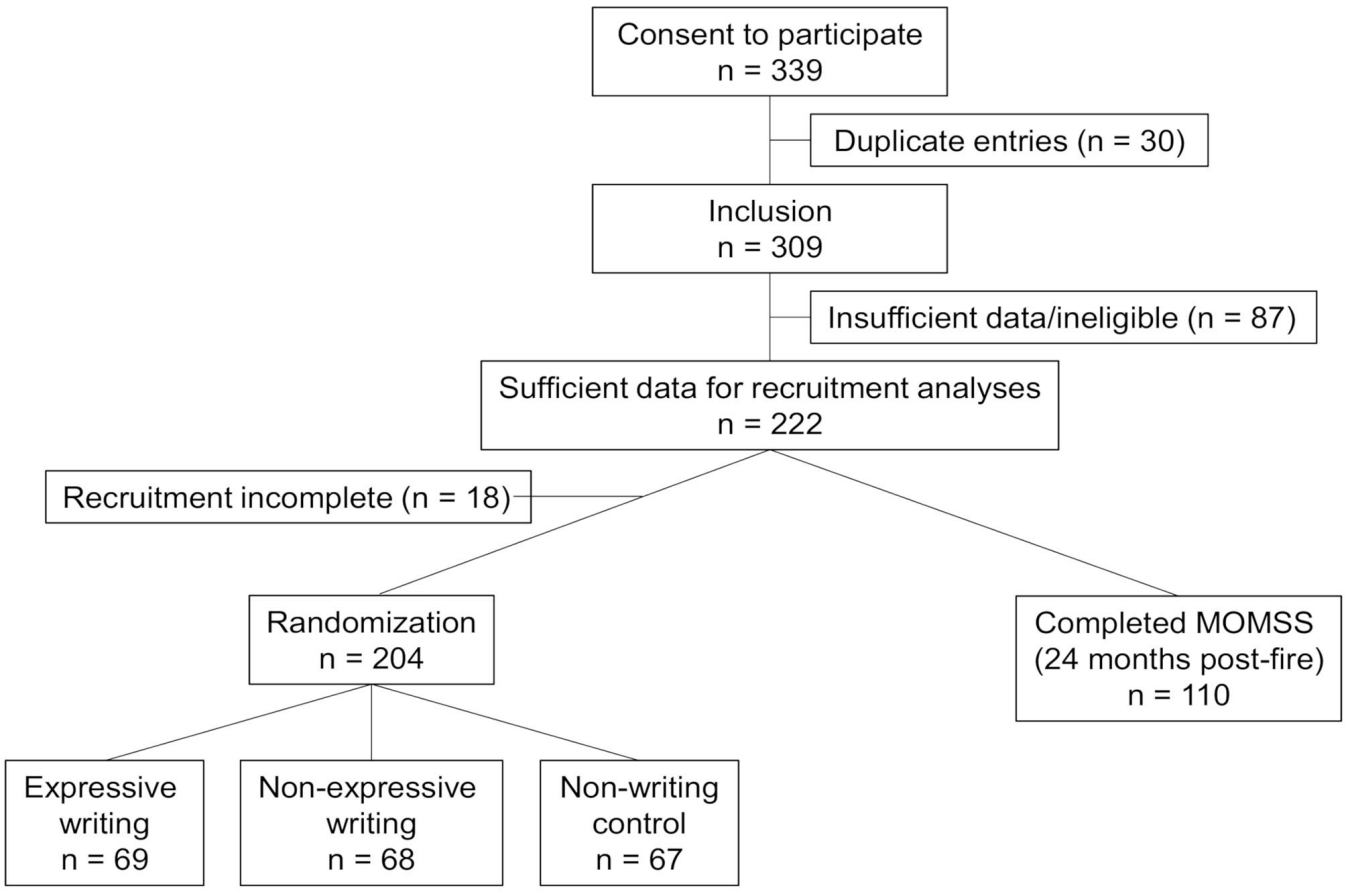
Flow chart illustrating the sample size and attrition in the Fort McMurray Mommy Baby study. Completion of all recruitment questionnaires was not a requirement for inclusion in at least part of the data analyses but was necessary for randomization for the writing intervention into the expressive writing group, non-expressive writing group, and non-writing or control group. The number of participants who completed the McMurray Objective Maternal Stress Scale (MOMSS) approximately 24 months after the fire is indicated.

Of the 339 records in the database, 309 were individual participants of which 222 participants filled out sufficient recruitment questionnaires to be included in at least part of the analyses. The other records were removed because of ineligibility or not containing sufficient data. A total of 204 women completed the recruitment stage and were randomized for the expressive writing intervention. The numbers of participants in each of the intervention and control groups were as follows: 69 participants in the expressive writing group, 68 participants in the non-expressive writing group, and 67 in the control group. Of these, 110 ultimately completed the objective stress questionnaire 24 months after the fire (Figure 1).

### Consent and Ethical Approval

This research protocol was reviewed and received ethical approval from the Health Research Ethics Board Health Panel at the University of Alberta (PRO-000067510). Eligible women were given information outlining the study purpose, benefits and risks of participating, and methods for withdrawal, prior to consenting to participate. They were also provided telephone contact information for the study coordinator should they wish to ask further questions about the study.

### Data Storage and Privacy

All data are stored in an online secure REDCap (Research Electronic Data Capture) database, hosted by the Women and Children's Health Research Institute at the University of Alberta, Edmonton, AB, Canada (86). Except for the 18-month postpartum evaluation, study consent, questionnaires, and the intervention were delivered electronically, although participants were given the opportunity to complete the MOMSS questionnaire by phone or to receive a paper copy in the mail. Data from the face-to-face assessment was entered manually. Upon completing the consent form, women were asked to supply their name, email address, and date of birth. This information was used to automatically send personalized invitations to complete the surveys and as login information for the participants for the questionnaires. Personal identifying information was solely available within the REDCap database and only accessible by selected team members. For analyses, the REDCap data were converted into Microsoft Word, Microsoft Excel, and SPSS documents using anonymous participant identifiers and date shifting, which were stored on a separate secure research drive and/or password protected.

### Intervention

After completing the recruitment questionnaires, participants were randomized for the expressive writing intervention into one of three writing groups as discussed above. The two active writing groups (expressive and non-expressive) were asked to write each day for four consecutive days with the following instructions: “In narrative form, please write constantly without stopping for 15 minutes about the topic. Provide as much factual detail and description as possible.” After each writing session, participants were asked to indicate to what extent, on a scale from 1 to 4, they were experiencing stress-related symptoms and emotions. Invitations to complete the writing tasks were sent out via email, containing a link to the writing exercise. Participants were asked to complete one exercise per day within 24 h of receiving the email invitation. Supplementary Table 1 provides an overview of the questions and topics included in the intervention.

### Quantitative Measures

All women recruited into the Fort McMurray Mommy Baby study were administered the same questionnaires. Upon completion of the study consent, women were asked to fill in a demographics questionnaire providing socioeconomic data (parental education, job classification, and estimated income), relationship status, obstetric history, and delivery date of the index pregnancy. The study protocol also included psychometric questionnaires as discussed below. An overview of the timing of the questionnaires is available in Table 1.

**Table 1:** Fort McMurray Mommy Baby Study data collection tools and timing.

**Measures**	**Recruitment**	**4 months postpartum**	**12 months postpartum**	**18 months postpartum**	**24 months post-fire**
**Maternal assessment questionnaires**					
Subjective stress (IES-R)	**X**				**X**
Peritraumatic Dissociation (PDEQ)	**X**				
Peritraumatic Distress (PDI)	**X**				
Resilience (CD-RISC)	**X**				**X**
Depression (EPDS)	**X**	**X**	**X**		**X**
State Anxiety (STAI-State)	**X**	**X**	**X**		**X**
Coping Style (Brief COPE)	**X**				**X**
Perceived Stress (PSS)	**X**	**X**	**X**		**X**
Social Support (SSQ)	**X**	**X**	**X**		**X**
Life Events (LES)		**X**	**X**		**X**
Obstetric and neonatal history questionnaire (87)		**X**			
Adverse Childhood Experiences (ACE)					**X**
Objective Stress (MOMMS)					**X**
Resilience statement					**X**
Cognitive appraisal					**X**
**Child assessment questionnaires completed by parent**					
Developmental milestones (ASQ)		**X**	**X**		
Language development (MB-CDI)			**X**		
Social and emotional development (BITSEA)		**X**	**X**		
**Child assessment: face-to-face**					
Development (Bayley III)				**X**	
Solo and Joint Free Play Protocol				**X**	
Height and weight				**X**	
Anthropometric measures and body composition				**X**	

*ACE, Adverse Childhood Experiences; ASQ, Ages and Stages Questionnaire; Bayley III, Bayley-III Scales of Infant and Toddler Development; BITSEA, Brief Infant Toddler Social and Emotional Assessment; CD-RISC, Connor-Davidson Resilience Score; COPE, Coping Orientations to Problems Experienced; EPDS, Edinburgh Postpartum Depression Scale; IES-R, Impact of Events Scale – Revised; LES, Life Experiences Survey; MB-CDI, McArthur-Bates Communicative Development Inventory; MOMSS, McMurray Objective Maternal Stress Score; PDEQ, Peritraumatic Dissociative Experiences Questionnaire; PDI, Peritraumatic Distress Inventory; PSS, Perceived Stress Scale; STAI, State-Trait Anxiety Inventory; SSQ, Social Support Questionnaire*.

#### Wildfire-Related Questionnaires

##### Objective Stress (24 Months Post-fire)

The degree of objective hardship due to the fire was measured by a questionnaire tapping into four categories of trauma exposure: Threat (e.g., threat to life or physical integrity), Loss (e.g., of persons or property), Scope (e.g., length of evacuation and interruption of communication), and Change (e.g., amount of displacement, change in routine). The McMurray Objective Maternal Stress Score (MOMSS) is based on similar questionnaires developed for previous natural disaster studies examining the objective stress experienced by participants in the Quebec ice storm as well as Iowa and Queensland floods (28–30). As this was the first post-wildfire population the team approached, a new questionnaire was developed. Because each disaster is different, the MOMSS includes questions about threat, loss, scope, and change that were tailored specifically to the FMWB wildfire. Responses on each category are scored to a maximum of 50 points/category for Change and Scope but −4 to 50 for Loss (−2/−4 for increase in household income in 2016), for a total possible score of 200. For Threat, a possible 5 additional points were given for injuries to partner and/or children and for witnessing flames touching the mother's own home. A fifth category, Thwart, consists of elements impeding or slowing the mother's ability to leave the city as well as external barriers to get all family members to safety, but was not included in the overall score. The invitation to complete the questionnaire was sent out 24 months after the fire to all women who had completed recruitment questionnaires.

##### Peritraumatic Distress and Dissociation (Recruitment)

Subjective distress is at least in part determined by peritraumatic distress and dissociation. Peritraumatic distress, assessed with the 13-item Peritraumatic Distress Inventory (PDI), is the degree of distress experienced at the time of or immediately after the disaster as recalled at a later point in time (88). Peritraumatic dissociation on the other hand is measured using the 10-item Peritraumatic Dissociative Experiences Questionnaire (PDEQ) and estimates the severity of dissociative-like experiences such as amnesia, derealization, depersonalization, altered perception of time, and out-of-body experiences (89). Both questionnaires are scored on a 5-point Likert scale from “Not at all” to “Extremely true.” Together, these measures serve as a predictor of which participants may be at increased risk of developing posttraumatic stress disorder (PTSD).

##### Subjective Stress (Recruitment, 24 Months Post-fire)

The Impact of Event Scale-Revised (IES-R) is used to assess the severity of PTSD symptoms in relation to traumatic events, as experienced in the 7 days preceding completion of the questionnaire (90). This 22-item questionnaire, with scores ranging from “Not at all” (0) to “Extremely” (4), yields a total score as well as scores for three categories of PTSD symptoms: intrusive thoughts, avoidance, and hyperarousal. This particular scale was chosen for our study as it enables comparisons to previous disaster studies including Project Ice Storm, the Iowa Flood Study, and Queensland Flood Study (35, 91, 92).

#### Maternal Psychological Health, Psychosocial Factors, and Other Maternal Measures

##### Maternal Anxiety (Recruitment, 4 and 12 Months Postpartum, 24 Months Post-fire)

Maternal anxiety was measured using the State-Trait Anxiety Inventory (STAI) (93), which asks participants how they generally feel, i.e., trait anxiety such as “I am content; I am a steady person,” or currently feel, assessing state anxiety with statements including “I feel tense; I feel frightened,” and is rated on a 4-point Likert scale from “Almost Never” to “Almost Always.” Internal consistency and test-retest reliability coefficients have been shown to range between 0.69 and 0.95 (93, 94).

##### Depression (Recruitment, 4 and 12 Months Postpartum, 24 Months Post-fire)

The Edinburgh Postnatal Depression Scale (EPDS) was used to measure maternal depression (95, 96). The EPDS is a 10-item questionnaire that indicates whether a woman has depressive symptoms including guilt, sleep disturbance, low energy, and suicidal ideations. It has been validated for antenatal and postpartum mothers as well as in women who have delivered more than 1 year prior to administration (95, 97). In English-speaking mothers, a cut-off score of 13 or more is suggested for probable major depression in postpartum women whereas 15 or more may be used antenatally (98). The EPDS, rated on a 4-point Likert scale (0–3), has high test-retest reliability and good internal consistency (Cronbach's alpha > 0.8) (99).

##### Coping Style (Recruitment, 24 Months Post-fire)

The Coping Orientations to Problems Experienced (COPE) and the abbreviated version Brief COPE identify coping strategies participants use when experiencing stress (100). The latter is a 28-item questionnaire that measures the way participants have been coping with stress in their lives over the last month with answers ranging from “Not true at all” to “True nearly all of the time” (101). It assesses 14 means of coping such as self-distraction, denial, substance use, positive reframing, humor, religion, and acceptance, which can be categorized into emotion and problem-focused coping as well as dysfunctional coping (102).

##### Life Events (4 and 12 Months Postpartum, 24 Months Post-fire)

The Life Experiences Survey (LES) used in this study is a 24-item score, derived from the original 57-item self-report questionnaire designed to inventory life changes participants experienced in the last year (103). Here, we asked about life events since conception of the child in the study, outside of the wildfire. It lists life changes in love and relationships such as marital status, health including pregnancy, pregnancy complications, and major illness of self as well as death and illness of loved ones, work-related and financial changes, and crime-related events. In addition to indicating whether the event occurred, participants are asked to rate the perceived impact of the event on a 7-point Likert scale ranging from “Extremely Negative (−3)” to “Extremely Positive (+3).”

##### Resilience (Recruitment, 24 Months Post-fire)

The Connor-Davidson Resilience Scale-25 (CD-RISC) is a 25-item questionnaire that measures resilience by asking participants how they may have felt over the preceding month (60). The scale covers several aspects of resilience including self-efficacy, sense of humor, attachment to others, the ability to adapt to change, optimism, and faith rated on a 5-point Likert scale ranging from “Not true at all” to “True nearly all the time.” The total score is used in analyses with low scores indicating low levels of resilience.

##### Resilience and Cognitive Appraisal (24 Months Post-fire)

At the 24 month post-fire timepoint, participants were also asked to respond to the following statement: “When things go wrong in my life it generally takes me a long time to get back to normal,” scored from “Don't agree at all (1)” to “Agree a lot (5).” Thus, for this statement, a lower score indicates higher levels of resilience. At the same time, cognitive appraisal of the fire was assessed by a single question, as follows: “Taking into account all of the effects of the Fort McMurray and Alberta wildfires on you and your family, what would you say have been the overall consequences of the event?” The consequences were also rated on a 5-point scale, from “Very negative,” over “Neutral, there were no consequences at all” to “Very positive.”

##### Social Support (Recruitment, 4 and 12 Months Postpartum)

The Social Support Questionnaire (Short Form) (SSQ) is a 6-item survey that asks participants about both the social support they have available to them as well as their level of satisfaction with the support available (104). The SSQ is a two-part survey, with part 1 asking participants to name their social supports in specific circumstances, if any, and the relationship to them while the second part asks participants to indicate their satisfaction with these supports, ranging from “Very satisfied” (6) to “Very dissatisfied” (1).

##### Perceived Stress (Recruitment, 4 and 12 Months Postpartum, 24 Months Post-fire)

The Perceived Stress Scale (PSS) was designed to evaluate the extent to which certain situations in the participant's life were considered stressful and thus how these situations affect individual feelings and the levels of perceived stress (105). It is a 14-item measure asking about thoughts and feelings in the past month, rated on a 5-point scale ranging from “Never” to “Very often.” Multiple items are stated in a positive way, e.g., “how often have you felt that you were on top of things” and are reverse coded such that higher scores indicate higher stress levels.

##### Adverse Childhood Experiences (24 Months Post-fire)

The Adverse Childhood Experiences (ACE) questionnaire, consisting of 10 questions, asks participants about personal experiences of abuse (psychological, physical, and sexual), as well as dysfunctionality within the household including mental illness, criminal behavior, violence against the participant's mother (106). The number of ACEs have been associated with increased risk of preterm birth (25) as well as risk behaviors and disease in adulthood (107, 108).

#### Developmental Milestones

##### Infant Development (4 and 12 Months Postpartum)

The Ages and Stages Questionnaire (ASQ) is a 30-item survey that asks mothers to assess five domains of infant development: communication, gross motor, fine motor, problem solving, and personal-social (109). It allows for the evaluation of developmental progress across these areas and is intended to catch delays in young children. Mothers are supplied with age-appropriate questionnaires and asked to answer questions using a 3-point Likert scale ranging from “Yes” to “Sometimes” to “Not yet.” At the end of the questionnaire, a list of overall questions is included assessing concerns regarding development noted by the parents. Examples are “Has your baby had any medical problems in the last several months?”, “Do you have concerns about your baby's behavior?” and “Does anything about your baby worry you?”. In the current study the ASQ-III 6 months and ASQ-III 12 months were used (110).

##### Language Abilities (12 and 18 Months Postpartum)

The MacArthur-Bates Communicative Development Inventories (MB-CDI) are instruments for assessing the communicative skills of infants (Words and Gestures) and toddlers (Words and Sentences) (111). We used the Infant form (Level 1), intended for 8- to 18-month-old children, limited to an 89-word vocabulary checklist for parents to indicate words that their child “understands” or “says.”

##### Social-Emotional Development (4 and 12 Months Postpartum)

The Brief Infant- Toddler Social Emotional Assessment (BITSEA) is a 42-item questionnaire that asks mothers about social-emotional and behavioral competencies and problems in their infants (112). It assesses behavior as observed over the last month, scored on a 3-point Likert scale (“Not true/rarely; Somewhat true/sometimes; Very true/often”). In addition, parents are asked to respond to two questions with possible answers ranging from “Not at all” to “Very worried,” i.e., “How worried are you about your child's behavior, emotions, and relationships?” and “How worried are you about your child's language development?” The different areas evaluated are then combined into two separate scales. The Competencies scale covers 11 items reflecting social-emotional abilities (e.g., empathy, imitation/play skills) whereas the Problems scale reflects internalizing and externalizing problems, maladaptive and atypical behaviors as well as dysregulation. A third scale, the BITSEA Autism score, combines 19 problem and competence items associated with Autism Spectrum Disorders (113, 114).

#### Face-To-Face Assessments: Mother and Child (18 Months)

##### Anthropometric Measures and Body Composition

At the children's age of ~18 months, parents were offered a face-to-face assessment with a trained experimenter. The child's body composition was evaluated, examining height, weight, head circumference as well as anthropometric measurements including mid-upper arm, waist, and calf circumferences, and triceps and subscapular skinfolds. In addition, maternal height, weight, and pregnancy status were recorded.

##### Cognitive and Motor Functioning

Cognitive, fine and gross motor abilities were assessed using the Bayley-III Scales of Infant and Toddler Development (115). Cognitive development is assessed through examination of thinking and problem solving, while motor development is assessed through examination of fine and gross motor skills.

##### Play Levels, Emotional Availability, and Attachment Relationships

During the face-to-face sessions, these were assessed and videotaped.

### Analytical Methods

#### Qualitative Analysis

We conducted an analysis of study participants' expressive writing entries using qualitative methodology to elucidate both their resilience factors and the effectiveness of expressive writing on their resilience. Findings from this qualitative analysis were compared and contrasted with data from other study measures including the Connor-Davidson Resilience Scale (CD-RISC) (60) and with qualitative data from other disasters. Since the expressive writing intervention was administered electronically only using online journals, transcription for data analysis was not required. The Word documents containing the writing entries linked to a numeric participant identifier were uploaded into NVivo 12 (QSR International, Melbourne, Australia) to facilitate coding according to the six phases of thematic analysis (116). These are (1) familiarization; (2) coding; (3) theme generation; (4) review of themes; (5) theme definition and naming; and (6) writing up with data analysis using an inductive approach.

#### Quantitative Data Analysis

All quantitative data are/were analyzed using Microsoft Excel and IBM SPSS (IBM Corp, Armonk, NY, USA) after thorough data cleaning and checking. Imputation of missing values was performed using the expectation-maximization method (117). The timing of the fire in relation to the stage of pregnancy at exposure (preconception, first, second, and third trimester), was determined by calculating the number of days between the start of the fire (May 1, 2016) and the best estimated due date. Preconception exposure was defined as having a due date more than 280 days after the fire and evacuation. First trimester exposure corresponded to due dates falling 187–279 days following May 1, second trimester between 94 and 186 days, and third trimester 0–93 days. Statistical tests conducted and planned for these data include demographic statistics using parametric and non-parametric tests according to normality of distribution and homogeneity of variance; Pearson and Spearman correlations; structural equation modeling; multiple linear regression with interaction terms in moderation models; repeated measures regression models. Timing of the fire will be/was included as one of the covariates in the moderation analyses. Significant interactions are further investigated using the PROCESS macro v3.4 for SPSS (118). Probing the interaction reveals the magnitude and significance of simple or conditional effects of a predictor according to the level of the moderator. Furthermore, using the Johnson-Neyman procedure, it indicates the region of significance, the moderator transition points at which the conditional effects of the predictor achieve or lose significance. The coordinates provided by PROCESS can then be used to graph interaction figures.

#### Dissemination

Data collection was finalized by October 2018, with data analyses ongoing. During the study, preliminary results were shared with the FMWB community as well as policy makers on several occasions. Data resulting from this study have been presented at local, national, and international meetings. Two papers have been published (119, 120) while several others are under review or in preparation.

## Discussion

The Fort McMurray Mommy Baby study was designed to test the effectiveness of an expressive writing intervention on a vulnerable population, i.e., pregnant and preconception women exposed to a natural disaster, and its effects on maternal, birth, and child developmental outcomes. We employed a low-cost intervention that was widely deployed electronically or by traditional mail. It required a small commitment in terms of time, physical or mental involvement from participants over 4 days. This was enough input to allow for emotional disclosure, the active ingredient of the intervention. As time passes for the participants, the tool could be re-used by them to manage stress and build personal resilience. Since upwards of 1.5 billion people have been affected by natural disasters in the last decade alone (121), it is more than likely that the need for such tools will increase. This is imperative to assure the health of future generations.

### Strengths and Challenges

In the development and execution of this protocol for a prospective randomized controlled trial in the aftermath of a natural disaster, we faced many challenges. While we were able to overcome some, others were insurmountable. Nevertheless, we feel it is important to share the lessons we learned so disaster research and the development of interventions for preconception and pregnant women and their children can move forward, even in these challenging research environments.

#### Strengths of the Study

Throughout this study, we employed community-based participatory research principles (122) which acknowledged the unique knowledge needs of the FMWB community, the need for involvement throughout the research process, and importance of dissemination of study findings on an ongoing basis. In the months following the wildfire, prior to establishing formal study funding, we met with several leaders within the community to co-design the study protocol including participant recruitment and retention strategies. We also invited a participant, a local mother who was pregnant during the wildfire, to be a member of our research team. She provided valuable insight into the social structure of the community, suggestions on recruitment strategies, and feedback on the study measures. Furthermore, despite the extensive distance between the research institution and the community (450 km), the research team made several trips to refine recruitment strategies and monitor the progress of the study. We also organized “forums” for key stakeholders in the community (i.e., study participants, policy makers, and community leaders) to share preliminary findings, strengthen the relationship with the community, and inquire about any modifications that needed to be made to the protocol.

In addition to the strong relationships with the FMWB community that were built during the development of the protocol, other strengths of this study included the rapid recruitment of eligible participants, low-cost intervention and data acquisition, and successful testing of the intervention. We were able to recruit approximately one-sixth (*n* = 309) of all estimated eligible women (*n* = 1,850) who were pregnant or within 6 months preconception at the time of the wildfire. As there were a number of other studies occurring within the community during the same time frame and our participants experienced significant disruptions to their personal lives with many leaving the geographical area, we consider this sample size and recruitment successful given the circumstances. The use of REDCap limited the costs associated with the collection of the study data. As well, the expressive writing intervention was simple and low-cost largely because it was administered via REDCap. Given the university's extensive experience with REDCap, the establishment of the database for the Fort McMurray Mommy Baby Study facilitated the rapid rollout of a subsequent disaster study (Hurricane Harvey in Texas, USA). A final strength of this study involved the successful testing of an expressive writing intervention in the context of a natural disaster. To our knowledge, this had not been previously carried out.

#### Challenges of Disaster Research

What makes populations who have gone through natural disasters interesting, i.e., experiencing a sudden severe stressor in a quasi-random manner that impacts a large community, inherently also creates limitations. Protocols for a post-disaster study are unique and, in most cases, not immediately available. Developing the protocol, acquiring funding, and setting up the study takes time, which causes delays in data collection and introduces the risk of recall bias. Furthermore, due to the nature of disasters, such protocols cannot be executed in controlled environments. Even when carefully planned, actually carrying out the study is difficult, not in the least because of the time-sensitivity. In fact, the circumstances of the Fort McMurray Wood Buffalo wildfire and its aftermath required us to modify the protocol and be flexible and creative as the project unfolded in order to ensure sufficient quality data collection. Adequate funds only became available ~one year after the fire. As such, we were not able to hire a research coordinator and research assistant, take on an aggressive recruitment strategy or offer incentives until Spring 2017. This, at least in part, resulted in a major revision of the envisioned sample size. Another adjustment that was required was a revision of the timeline to perform the in-person developmental assessment. In accordance with our previous disaster studies, this evaluation was initially planned for when the infants were 16 months old (30). Because of the delay in funding as well as difficulties in finding a qualified research nurse, this evaluation had to be postponed for 2 months. Moreover, we were only able to assess 33 children.

The objective stress questionnaire was distributed to the participants later than scheduled as well. As this was our first post-fire study, a new survey needed to be designed. The development of the questionnaire requires an in-depth understanding of the complex geographic, social, and economic environment, with a need for extensive examination and preparation. This was hindered by the fact that none of the members of the research team lived in the community as well as the distance.

The obvious need for quick development of a protocol and application for funding may also hinder the quality of the study in the long term. In the time frame available to design the study and apply for funding, it may not be possible to set up an optimal multidisciplinary research team or contact experts. This may lead to important questions not being asked. Another consideration is how participants interact with the online secure database because there is not enough time for beta testing. For example, some individuals participated more than once, and the database should have prevented this. A number of data entry mistakes required extensive data cleaning. This is an inherent problem when participants enter data on mobile devices. Also, email invitations are easy to overlook or forget as there is no physical reminder of the questionnaire. Participants received up to five reminder emails with the link to the appropriate questionnaire.

Careful thought also needs to be given to the time required to complete the questionnaires. The initial batch of surveys in this study took over 1 h to complete. Moreover, often when there is no immediate tangible benefit to participants, they will not continue completing the questionnaires. In an effort to minimize this problem and in recognition of the significant time commitment required to complete the surveys, we decided while the study was ongoing to compensate participants for their time with $50 gift cards for online purchases. To further encourage completion of the questionnaires, the order of the surveys was changed, partly in recognition of the fact that several of the questions may have been perceived as difficult or sensitive and could have caused participants to drop out. Aside from the number and length of the questionnaires, another element to keep in mind is to not overly study the community undergoing significant stress as this can lead to research fatigue. At the time of the current study, multiple others were being conducted, several funded through the same government agency, each with their own demands. When individuals participate in different studies at the same time, it may be possible that these demands increase stress and that potential positive effects of participation may be canceled out.

In addition, disentangling the effects of exposure to a disaster from the alterations in mental health that are inherently associated with the reproductive period without overwhelming participants with numerous questionnaires is very difficult. Although pregnancy and childbirth are physiological processes and not illnesses, they can be associated with negative psychological experiences related to unexpected pregnancy, loss of a pregnancy, challenging obstetric history, and maternal and fetal complications. Moreover, one needs to consider other traumatic events in a woman's life, ranging from adverse childhood experiences to other life events and difficult environmental circumstances, i.e., racism, violence, unemployment, substance abuse, and socioeconomic struggles, prior to, during, and after pregnancy and childbirth (25, 123–125). The latter is especially the case in disaster research since recruitment is often only possible after a considerable time lag, during which much in addition to the disaster could have occurred in a woman's life. The Adverse Childhood Experiences questionnaire and Life Experiences Survey were included in the protocol but were only completed by a limited number of participants, preventing us from correcting for these events in analyzing the data.

### Limitations

A limitation of disaster-related studies is that the stress is not limited to the event itself; it continues to accumulate due to the consequences of the event and may therefore affect who participates in the study. This leads to two problems inherent in such studies: that some participants may not be able to have the time or the focus to participate in the study due to being overwhelmed by stress, and that it may be the least affected by stress who are disproportionately represented in the study. Ongoing stress may derive from temporary housing or relocation issues, fear for the safety, health, and well-being of loved ones, economic and/or food insecurities, and in the case of the FMWB disaster, longer-term disputes with insurance agencies.

### Recommendations

Our recommendations for future studies that test interventions to improve pregnancy outcomes in the midst of disasters are: (1) recruit in larger communities where many more participants live so that the power of the study is maintained in spite of the participant attrition due to all the reasons described here. (2) Recruit the most stressed participants into your study in order to create a robust effect size so that your intervention has the largest effect possible. For instance, during the current coronavirus pandemic there are many pregnant women, some of whom are highly stressed. Focus your recruitment on those you consider to be the most stressed. (3) Prepare in advance for a disaster intervention study. It is possible for investigative teams to obtain ethical approval (or at least in a draft form), develop their protocols, interventions, and database tools in advance of a natural disaster. Then, when a significant disaster strikes a community, the team can quickly implement their study. The costs for this preparedness are surprisingly low; it is the analysis phase of the study that is more expensive due to research staff costs. However, once the data are gathered, it is easier to obtain funding for these analyses.

## Conclusion

The first purpose of this paper was to describe the development of a protocol to test an intervention offered to pregnant and preconception women following a disaster. The Fort McMurray Mommy Baby Study was the first study attempting to reduce stress in preconception and pregnant women after a catastrophic natural disaster. Previous studies largely documented the degree of stress women experiencing a natural disaster had and the effects of that stress on their pregnancy outcomes and the developmental trajectories of their children. Similar studies evaluate the impact of these events at both the community and the individual level, but none have attempted to reduce stress, increase resilience, and thereby improve outcomes. These are the problems the research in this field needs to address.

The second purpose was to outline the challenges encountered in the context of this research. As a first study in this regard, we experienced several challenges. These included the *a priori* study design not conforming to the realities of a post-disaster environment in a remote community. There were challenges with participant recruitment and retention that led to consequent loss of data, reduced sample size, and gaps in the data. There might also have been selection and recall biases. Last, even though we started organizing the study as soon as the disaster occurred, it took 6 months to obtain ethical approval, funding, and to initiate the study. This time lag may have led to some of the problems encountered. Fortunately, this is an iterative process whereby improvements will occur with repeated attempts to respond to future natural disasters with studies that test interventions to improve outcomes.

## Data Availability Statement

The original contributions presented in the study are included in the article/Supplementary Material, further inquiries can be directed to the corresponding author.

## Ethics Statement

This study involving human participants was reviewed and approved by Health Research Ethics Board Health Panel, University of Alberta. The participants provided their written informed consent to participate in this study.

## Author Contributions

Each author contributed to the design of the study. AH drafted the manuscript, which was revised and completed by AH, BV, JO, and DO. All authors contributed to the article and approved the submitted version.

## Conflict of Interest

The authors declare that the research was conducted in the absence of any commercial or financial relationships that could be construed as a potential conflict of interest.

## Footnotes

^1^www.mommybabyfmm.com.
