# Supplementary material for: The Fort McMurray Mommy Baby Study: A Protocol to Reduce Maternal Stress Due to the 2016 Fort McMurray Wood Buffalo, Alberta, Canada Wildfire
Source: Front Public Health. 2021 Jun 17;9:601375. doi: 10.3389/fpubh.2021.601375 (PMC8249202; doi:10.3389/fpubh.2021.601375)
Supplement: Supplementary file 1 [file Table_1.DOCX]

Supplementary Table 1: Writing prompts and reflections administered to the expressive and non-expressive writing groups. Participants were asked to write for 15 minutes without interrupting on 4 consecutive days as follows: “In narrative form, please write constantly without stopping for 15 minutes about the topic. Provide as much factual detail and description as possible.”

|  | Day 1 | Day 2 | Day 3 | Day 4 |
| --- | --- | --- | --- | --- |
| Expressive writing questions | What are your worst fears due to the fire?  What has helped you deal with those worst fears? | Has the wildfire caused any changes in your personal relationship(s) with another person or persons?  What is (are) the worst example(s)?  What has helped or is helping you deal with these relationship changes? | What is the most traumatic, upsetting experience of your entire life, especially that you have never discussed in great detail with others?  What helped you deal with this experience? | What are the worst conflicts or problems that you have ever experienced or are experiencing now?  What helped you deal with this situation? |
| Non-expressive writing topics | Exercise | Diet | General health | Work |
| Daily reflection | For each of the items, please indicate to what degree (between 1 and 4) you are currently experiencing the following:   \| Racing heart  Upset stomach  Headache  Dizziness \| Shortness of breath  Cold hands  Sweaty hands  Pounding heart \| Nervous  Sad  Guilty  Happy \| Contented  Fatigued  Constrained  Anxious \| \| --- \| --- \| --- \| --- \| | | | |
